# The Accessory Molecule Lgp55 Plays a Role Early in Murine Fetal Thymocyte Differentiation

**DOI:** 10.1155/1994/29157

**Published:** 1994

**Authors:** Marcia Mcduffie, William T. Golde

**Affiliations:** 1 Department of Pediatrics University of Virginia Health Sciences Center Charlottesville Virginia 22908 USA; 2 Center for Vector Borne Infectious Diseases Centers for Disease Control Fort Collins Colorado 80522 USA

**Keywords:** T cells, differentiation, accessory molecules, thymus

## Abstract

A rat IgM monoclonal antibody, PA3-795, inhibits the antigen-specific responses of mouse T-cell hybridomas. It recognizes a heavily glycosylated cell-surface protein, designated Lgp55, that is detectable after activation on mature T cells. During fetal life, Lgp55 is found at high levels on newly immigrant thymic T-cell precursors prior to surface expression of other T-lineage molecules. High levels of expression are also found on thymocytes in the outer cortex of adult mice. Thymocytes at later stages of differentiation bear decreasing amounts of surface Lgp55, and none is detectable on “single-positive” thymocytes in the thymic medulla or on resting mature T cells from the periphery. Addition of monoclonal anti-Lgp55 to fetal thymus organ culture decreases the output of “mature” CD4 singlepositive thymocytes when it is begun before fetal day 13.5. These findings suggest that Lgp55 contributes to cell-cell interactions that regulate very early steps in T-cell development in the mouse.


*Developmental Immunology*, 1994, Vol 3, pp. 257–263
Reprints available directly from the publisher
Photocopying permitted by license only

© 1994 Harwood Academic Publishers GmbH
Printed in Singapore

### The Accessory Molecule Lgp55 Plays a Role Early in Murine Fetal Thymocyte Differentiation

MARCIA MCDUFFIE*[†] and WILLIAM T. GOLDE[‡]

[†]*Department of Pediatrics, University of Virginia Health Sciences Center, Charlottesville, Virginia 22908*
[‡]*Center for Vector Borne Infectious Diseases, Centers for Disease Control, Fort Collins, Colorado 80522*

A rat IgM monoclonal antibody, PA3-795, inhibits the antigen-specific responses of mouse T-cell hybridomas. It recognizes a heavily glycosylated cell-surface protein, designated Lgp55, that is detectable after activation on mature T cells. During fetal life, Lgp55 is found at high levels on newly immigrant thymic T-cell precursors prior to surface expression of other T-lineage molecules. High levels of expression are also found on thymocytes in the outer cortex of adult mice. Thymocytes at later stages of differentiation bear decreasing amounts of surface Lgp55, and none is detectable on "single-positive" thymocytes in the thymic medulla or on resting mature T cells from the periphery. Addition of monoclonal anti-Lgp55 to fetal thymus organ culture decreases the output of "mature" CD4 single-positive thymocytes when it is begun before fetal day 13.5. These findings suggest that Lgp55 contributes to cell–cell interactions that regulate very early steps in T-cell development in the mouse.

KEYWORDS: T cells, differentiation, accessory molecules, thymus.

### INTRODUCTION

Undetectable on resting peripheral mouse T cells, Lgp55 is expressed at high levels on activated T cells (both CD4 and CD8) and most T-cell hybridomas. Binding by monoclonal anti-Lgp55 (PA3-795) inhibits low-affinity responses of T-cell hybridomas to antigen presented by B-cell lymphomas (Glode et al., 1990). Anti-Lgp55 also inhibits proliferation and the generation of cytotoxic lymphocytes (CTL) in primary mixed-lymphocyte reactions *in vitro* (Golde et al., 1990; M. McDuffie and R. Gill, unpublished data). Thus, the functional evidence to date indicates a role in the immune response similar to that of the adhesion molecule LFA-1 and its ligands (Davignon et al., 1981; Boyd et al., 1988). Like mature T cells after activation, most adult thymocytes (> 50%) express Lgp55 at moderate to high levels (Golde et al., 1990). This finding raised the possibility that Lgp55 might enhance interactions between T-cell precursors and the thymic stromal cells that direct the developmental sequence.

During murine fetal development, lymphoid precursors from yolk sac and fetal liver begin to colonize the murine epithelial thymus structure on days 11 and 12 of the 19- to 21-day gestation. On day 12, the thymus is well-colonized with relatively quiescent lymphoid precursors. By day 14 of gestation, these lymphoid precursors are actively proliferating, and the T-cell marker Thy-1 has appeared on a minority of thymocytes (Fig. 2; Ceredig et al., 1983). In addition, the earliest rearrangements of T-cell receptor genes have occurred (Born et al., 1986) and IL-2 message is detectable (Pardoll et al., 1987). By day 15, mature T cells of the $\gamma\delta$ type have developed, Thy-1 is expressed on the majority of lymphoid cells, and the differentiation of the first $\alpha\beta$ T cells, which will appear on day 18, is well underway. The signals that initiate T-cell development on day 13 have not been determined, but these steps do not occur to any significant degree in the absence of a functional thymic epithelium (Maryanski et al., 1981).

To determine the developmental stage in which Lgp55 might participate, thymocytes from fetal mice were first examined for expression of Lgp55 in relation to other markers of thymocyte differentiation. Because LFA-1 plays a role similar to Lgp55 on

*Corresponding author.

mature T cells and is also expressed on the majority of thymocytes in the adult mouse (Golde et al., 1990), its expression was examined along with that of Lgp55. Detection of high levels of Lgp55 on early thymic presursors, in advance of other T-cell markers, prompted and exploration of the possible role of this accessory molecule in T-cell differentiation.

### RESULTS

Both LFA-1 and Lgp55 were coexpressed on most thymocytes from fetal mice at day 15 of gestation (data not shown). However, the thymocyte precursors found at day 13 expressed only Lgp55 (Fig. 1, panel A). LFA-1 expression first appears on a significant proportion of Lgp55$^+$ thymocytes between days 13 and 14 of fetal development. During the transition from day 14 through day 16, increasing percentages of thymocytes coexpress Lgp55 and LFA-1 (Fig. 1, panels B–D). This period coincides with rearrangement of all T-cell receptor genes, ending with rearrangement of the $\alpha$-chain gene and the first appearance of functional $\alpha\beta$ T-cell receptor molecules on the surface of the developing thymocytes. From the end of day 16 to adulthood, an increasing percentage of the thymocytes displays the LFA-1$^{high}$/Lgp55$^{low-moderate}$ phenotype, reaching 85% in the adult (Fig. 1, panel E). This subpopulation of thymocytes must be largely coincident with, if not identical to, the bulk of nonmature CD4$^+$/CD8$^+$ "double-positive" cells that also comprise ≈ 85% of the cells in the adult murine thymus and serves as an intermediate step in the production of $\alpha\beta$ T cells. Simultaneously, the fraction of LFA-1$^-$/Lgp55$^{high}$ cells falls from > 90% early on day 14 to ≈ 1% in the adult.

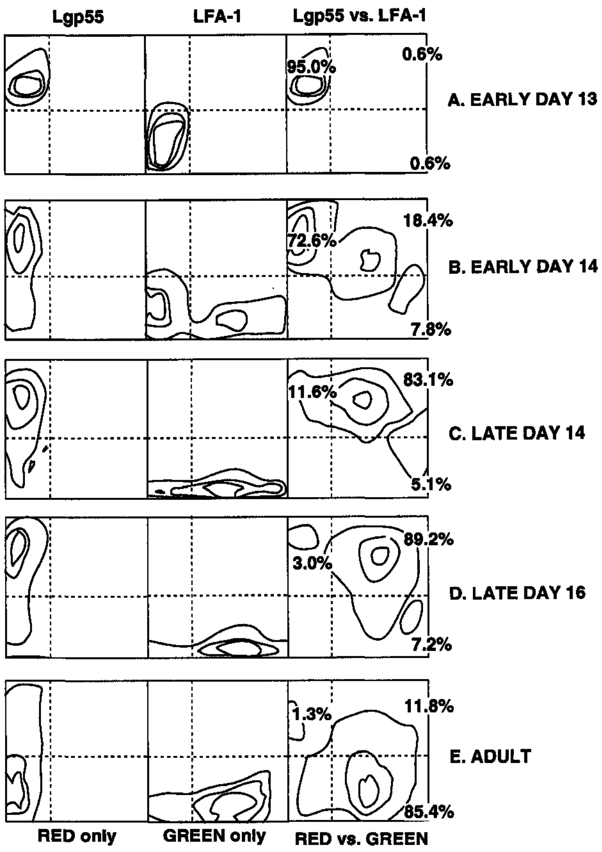

FIGURE 1. Fetal C57BL/6J mice from timed pregnancies were removed on the indicated day of gestation, and thymocyte suspensions from one litter for each gestational age or from a single adult were analyzed for expression of Lgp55 and LFA-1. Lgp55 was detected using biotinylated PA3 antibody (monoclonal rat IgM); Streptavidin-phycoerythrin (PE-SA); LFA-1, using monoclonal rat IgG I21/7.7 as culture supernatant; and a fluorescein-conjugated goat anti-rat IgG Fc (FITC-GAR Fc) that does not cross-react with IgM. Ten thousand cells were counted for samples from day 14; $2.5 \times 10^4$ for day 16; and $4.0 \times 10^4$ for adult. Data shown are representative of three separate experiments.

Like LFA-1, early markers of precursor commitment to T-cell lineages, such as Thy-1 and the low-affinity p55 IL-2 receptor (IL-2R) are also expressed on the Lgp55$^+$ thymocyte subpopulation. Figure 2, panel A, shows the progression of increased expression of Thy-1 from day 14 to 17 of fetal development: Virtually all Thy-1$^+$ cells in the day-14 fetal thymus coexpress moderate to high levels of Lgp55. Two-color immunofluorescence confirms that these cells also bear LFA-1 (data not shown). The IL-2R$^+$ cells previously described by others as forming a significant subpopulation among thymocytes during these stages of maturation (Ceredig eg al., 1985; Raulet, 1985; Shimonkevitz et al., 1987) also express Thy-1, and, by inference, Lgp55 (Fig. 2, panel B) and LFA-1. In four separate experiments, Thy-1$^+$ cells harvested from early day-14 fetal thymus lobes ranged from 4 to 22% of the nucleated cells; IL-2R$^+$ cells, from 3 to 9%.

Monoclonal antibody staining of frozen sections from adult thymus confirmed the impression, based on flow cytometric analyses, that Lgp55 is expressed only on cortical thymocytes, whereas LFA-1 is present on thmocytes found in both cortex and medulla (data not shown). Absolute lack of staining by anti-Lgp55 on medullary thymocytes indicates that cells at this developmental stage are similar to

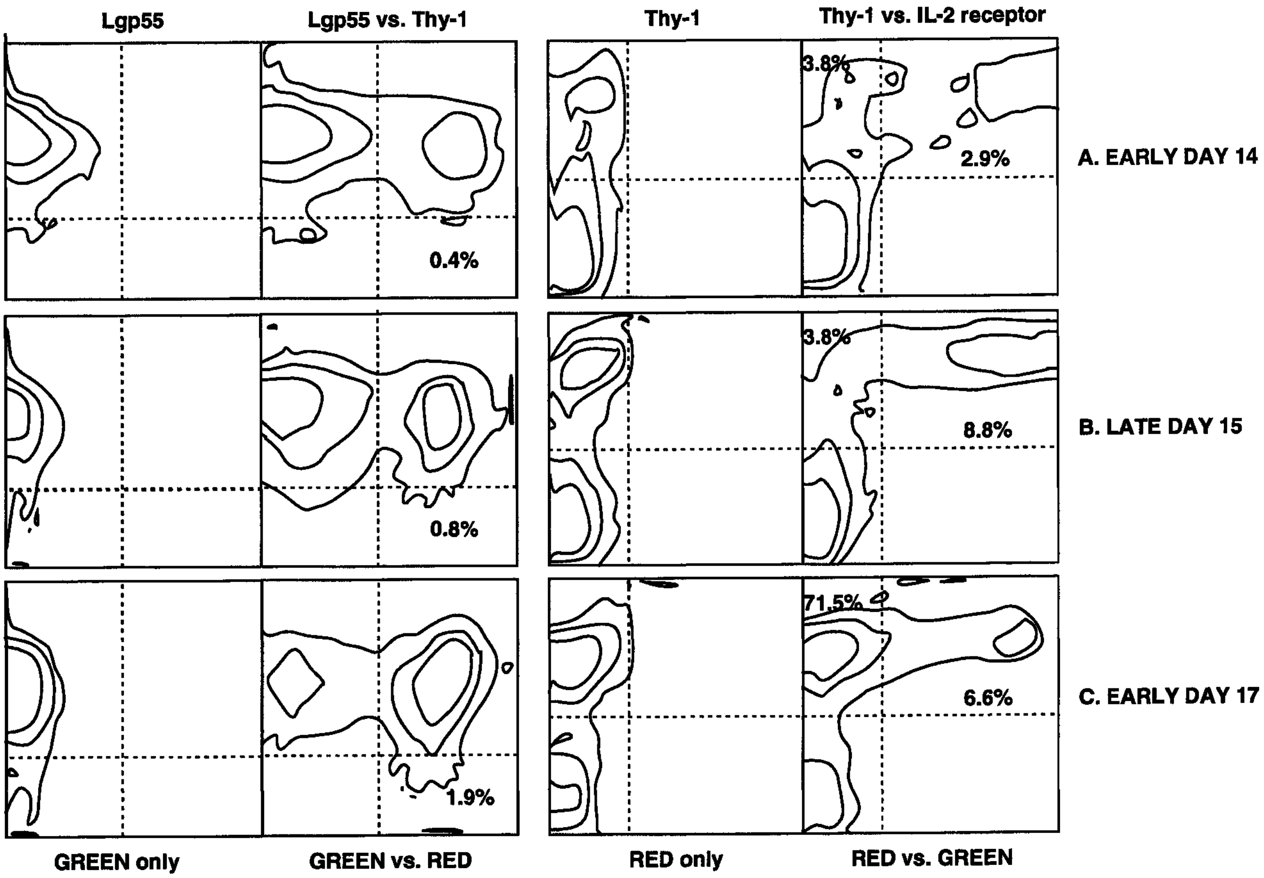

FIGURE 2. Samples for cytofluorographic analysis were prepared and analyzed as described for Fig. 1. Lgp55 was detected using PA3 culture supernatant and fluorescein-conjugated RG7/9.1; low-affinity IL-2 receptors (p55) with monoclonal rat PC61 supernatant and FITC-GAR Fc; Thy-1, using biotinylated T24/40.7 0 and PE-SA. For each primary antibody (PA3 or PC61), free binding sites on the secondary antibodies were blocked by a second incubation with supernatant containing the primary antibody prior to exposure to biotinylated anti-Thy-1. Results were confirmed using biotinylated PA3 and either T24/40.7 or PC61 supernatant in the same protocol as described in the legend to Fig. 1 for anti-LFA-1. Samples of $\geq 10^4$ cells were analyzed for each histogram. Data shown are representative of four separate experiments.

peripheral T cells, on which no detectable Lgp55 is expressed prior to activation.

The murine analog of human ICAM-1, shown by direct binding studies to be a ligand of LFA-1, is identified by the monoclonal antibody YN1/1.7. This molecule is not found on thymocytes bearing high levels of Lgp55 (Fig. 3A). ICAM-1 expression, therefore, does not mark the earliest thymic precursors and is unlikely to play a role in the earliest stages of differentiation. It may, however, have an impact on some subsets of thymocytes at later developmental stages.

Following commitment to the T-cell lineage during the first stage of thymocyte maturation, two pathways of differentiation appear in the fetal thymus by late day 16. Thy-1$^+$ cells bearing CD3 and $\gamma\delta$ T-cell receptors have developed and represent 3 to 6% of thymocytes. Simultaneously, thymocytes that have rearranged $\alpha$ and $\beta$ T-cell receptor genes begin to express CD8 molecules as they enter the phase of T-cell differentiation during which developing clones are "selected" for self-tolerance and restriction to self-MHC. Figure 4 shows that both CD8 and CD3 are expressed exclusively on Lgp55$^+$ thymocytes at day 16 of fetal development. Whereas the CD8$^+$ cells represent the major $\alpha\beta$ pathway of thymocyte differentiation, the CD3$^+$ cells at this stage are presumed to bear $\gamma\delta$ T-cell receptors, as they are unable to bind at T-cell receptor antibody, H57-597, which binds to all $\beta$ chains (Fig. 4C). Thus, both of the major pathways of thymic T-cell development derive from Lgp55$^+$ precursors.

To determine the role of Lgp55 in T-cell development, fetal thymus lobes from C57BL/6J mice were

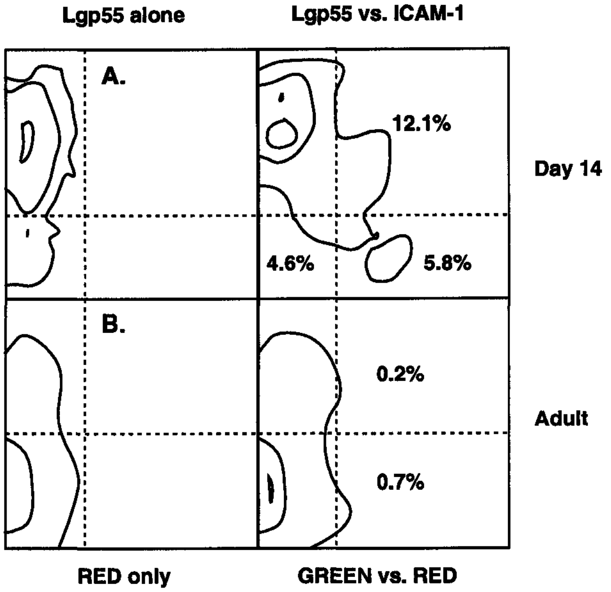

FIGURE 3. Thymocytes, pooled from a litter of fetal mice at day 14 of gestation or from a single adult, were incubated with biotinylated PA3, with or without ascites from the hybridoma YN1/1.7, diluted 1/100 in balanced salt solution. FITC-GAR Fc and PE-SA were used as secondary reagents. Ten thousand cells from day 14 fetal thymuses were analyzed, and $4 \times 10^3$ adult thymocytes.

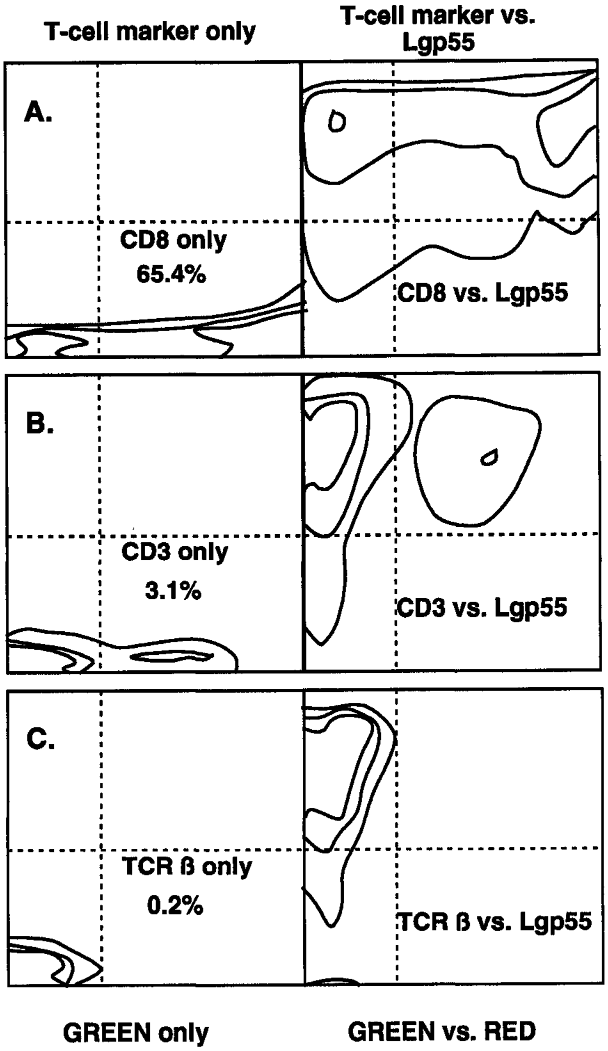

FIGURE 4. Aliquots of the thymocytes from the day-16 litter shown in Fig. 1 were analyzed for expression of CD8, CD3, and T-cell receptor $\alpha\beta$ using a staining protocol (with blocking for anti-CD3 and anti-$\alpha\beta$) as described in the legend for Fig. 2. Lgp55: biotinylated-PA3 with PE-SA; CD8: monoclonal 53-6.72 as supernatant and FITC-GAR Fc; CD3: monoclonal 145-2C11 and fluorescein-conjugated goat anti-mouse Ig that cross-reacts with rat Ig light chains (FITC-GAM); T-cell receptor $\alpha\beta$: monoclonal H57-597 and FITC-GAM.

cultured with anti-Lgp55 for periods of 2.5 to 6.5 days. Control cultures were treated with buffer alone (phosphate-buffered saline, [PBS], pH 7.4) or control antibodies. Table 1 summarizes three representative experiments using thymus lobes from mice at days 13 to 14 of gestational age cultured for 2.5 to 3 days. For these experiments, the control antibody used was M5/114, a monoclonal rat IgG that recognizes the I-$A^b$ molecule (Bhattacharya et al., 1981). This antibody binds to thymocytes via passively acquired I-A molecules (Sharrow et al., 1981), which may not be integrated normally into the cell membranes. Treatment with anti-Lgp55 decreased the cell numbers produced from test cultures compared to controls when added to thymus lobes harvested early on day 13 of gestation. Table 1 shows that thymus lobes from gestational ages of $< 13.5$ days were consistently more susceptible to the effects of anti-Lgp55 than those $\geq 13.5$ days in each individual experiment. Five additional experiments using pooled thymus lobes from litters of defined gestational ages confirmed these results for culture periods of 3 to 6.5 days (data not shown). Because interactions via $\alpha\beta$ T-cell receptors and MHC Class II molecules clearly play a role in survival of some thymocytes during the second stage of T-cell development (days 4 to 6 of fetal thymus organ culture), the control cultures for experiments using culture periods of $> 4.5$ days contained either PBS alone or a rat monoclonal anti-TCR idiotype (KJ1-26; Haskins et al., 1983) that binds to an undetectable number of cells in these thymus lobes.

Most importantly, anti-Lgp55 antibody treatment also reduces the number of phenotypically mature $CD4^+8^-$ in fetal thymus organ culture. Table 2

TABLE 1
Cell Yield Is Decreased in Anti-Lgp55-Treated Fetal Thymus Organ Culture

| Experiment | Fetal age (days) | Culture (days) | Treatment | Viable lymph. (× $10^3$/lobe) | % of control |
|---|---|---|---|---|---|
| 1[a] | 13 | 3 | Anti-Ia | 40.0 | |
| | | | Anti-Lgp55 | 0[b] | 0 |
| | 14 | 3 | Anti-Ia | 54.9 | |
| | | | Anti-Lgp55 | 27.3 | 50 |
| 2[a] | 13 | 3 | Anti-Ia | 31.9 | |
| | | | Anti-Lgp55 | 1.5 | 5 |
| | 13.5 | 3 | Anti-Ia | 70.1 | |
| | | | Anti-Lgp55 | 17.0 | 24 |
| | 14 | 3 | Anti-Ia | 49.2 | |
| | | | Anti-Lgp55 | 33.1 | 67 |
| | 14 | 3 | Anti-Ia | 74.4 | |
| | | | Anti-Lgp55 | 17.0 | 23 |
| 3[a] | 13 | 3 | Anti-Ia | 29.0 | |
| | | | Anti-Lgp55 | 12.7 | 44 |
| | 13.5 | 3 | Anti-Ia | 61.1 | |
| | | | Anti-Lgp55 | 60.9 | 100 |
| 4[c] | 13 | 3 | None | 9.2 | |
| | | | None | 9.6 | |
| | | | Anti-Ia | 10.6 | 113 |

[a]Lobes from single litters, dated by vaginal plug and by thymus morphology at the time of sacrifice, were divided equally between control and experimental culture wells.
[b]No viable lymphoblasts detected.
[c]Pooled thymus lobes from three litters.

TABLE 2
Anti-Lp55 Decreases Production of Mature T Cells

| Expt. | Fetal age | Treatment | Days of culture | $CD4^+/8^-$ ($\times 10^4$/lobe) | $CD4^+/8^+$ ($\times 10^4$/lobe) | $CD4^-/8^+$ ($\times 10^4$ lobe) |
|---|---|---|---|---|---|---|
| 1 | 13 | No Ab | 6 | 1.1 | 3.88 | 1.2 |
| | | Anti-Lgp55 | 6 | 0.15 | 0.7 | 0.4 |
| 2 | 13 | No Ab | 6 | 2.5 | 7.7 | 8.9 |
| | | No Ab | 6 | 1.7 | 9.4 | 11.5 |
| | | Anti-Lgp55 | 6 | 0.5 | 5.1 | 7.5 |
| | | Anti-Lgp55 | 6 | 0.7 | 5.8 | 7.4 |
| 3 | 14 | No Ab | 7 | 2.7 | 18.0 | 3.1 |
| | | Anti-Lgp55 | 7 | 1.1 | 9.2 | 2.4 |

shows the results of three experiments in which this was examined. In all experiments, these cells were decreased in number along with the total numbers of "immature" double-positives ($CD4^+/8^+$) and the mixed population of $CD4^-/8^+$, which consists of both double-positive precursors and mature cells.

### DISCUSSION

Thymic T-cell differentiation is an example of ongoing organogenesis in the mature mammal. In the thymic cortex, undifferentiated precursor cells from bone marrow proliferate and acquire surface expression of the interactive molecules necessary for mature T-cell function. Subsequent steps in differentiation lead to the selection of a self-restricted, largely self-tolerant T-cell repertoire. Cell–cell interactions mediated by receptor/ligand binding are known to play an integral role in the second or "selection" phase of T-cell differentiation in the thymus. For example, the "accessory" molecules CD4 and CD8, which bind to MHC molecules, participate in the process of clonal deletion that eliminates self-reactive T cells during the final stages of thymocyte differentiation (Kappler et al., 1987; Smith, 1987; Fowlkes et al., 1988; McCarthy et al., 1988).

Fine and Kruisbeek (1991) have suggested that ICAM-1 influences thymocyte differentiation. In their experiments, exposure to anti-ICAM-1 was shown to decrease the numbers of $CD4^+/8^+$ thymocytes during the early days of fetal thymus organ culture (days 3 and 4, after harvest at day 13 or 14

of fetal development). However, despite the transient decrease in "double-positive" cells, differentiation of $CD4^-8^+$ and $CD4^+8^-$ cells was not impaired. These authors also attempted to use PA3-795 for phenotypic analysis of thymocytes. Their antibody preparation did not bind to either fetal or adult thymocytes and, not surprisingly, did not influence thymocyte differentiation in fetal thymus organ culture. They attempted to isolate antibody from ascites using ammonium sulfate precipitation and column sizing appropriate for purifying IgG molecules. Because the PA3 antibody is a pentameric IgM that has no measurable affinity for Lgp55 in the reduced state (M. McDuffie, unpublished data), it is probable that the protein preparations used in this study contained no antibody molecules capable of binding the Lgp55 antigen.

Using our antibody preparations, Lgp55 can be detected at the highest levels on day-13 thymocytes. When day-13 fetal thymus lobes were exposed to antibodies inhibiting Lgp55 function, cell numbers in treated cultures were decreased from those in control cultures, whether the controls were untreated or bathed in medium containing an irrelevant rat antibody. Similar cultures of day 13.5 to 14 thymus lobes, in which thymocytes had already entered a committed phase of T-cell differentiation marked by expression of Thy-1 and p55 IL-2R, were more variably affected. These findings suggest that Lpg55 accessory function enhances the earliest steps of differentiation in the thymus, but is not required for continued differentiation once the initial signal has been transmitted. The lack of importance of Lpg55 in later stages of T-cell development is mirrored by decreasing levels of surface expression as the cells mature. Proliferation and differentiation have not been shown to occur in the absence of thymic stromal cells, so it is likely that the receptor for this molecule will be found on the thymic stromal cells that direct these steps.

### MATERIALS AND METHODS

### Mice

C57BL/6J mice were bred from stock obtained from the Jackson Laboratory (Bar Harbor, ME). The day of vaginal plugging was designated "day 0" of gestation for fetal mice. Adults used for cytofluorograph analyses were between 2 and 6 months of age.

### Antibodies

PA3-795.4.16 (monoclonal rat IgM, anti-Lgp55) was isolated from spleen cells of Sprague-Dawley rats, immunized with the DBA/2-derived mastocytoma P815, as previously described (Golde et al., 1990). The other antibodies used for cytofluorographic analyses were the following: I21/7.7 (rat monoclonal IgG, anti-LFA-1; I. Trowbridge), T24/40.7 (rat monoclonal IgG, anti-Thy-1; I. Trowbridge); PC61 (rat monoclonal IgG, anti-IL-2 receptor; Hashimoto et al., 1986); YN1/1.7 (rat monoclonal IgG, anti-ICAM-1; Prieto et al., 1989); 53-6.72 (rat monoclonal IgG, anti-CD8; Ledbetter and Herzenberg, 1979); 145-2C11 (monoclonal hamster IgG, anti-CD3ε; Leo et al., 1987); H57-597 (hamster monoclonal IgG, anti-TCR$\alpha\beta$; Kubo et al., 1989); and RG7/9.1 (monoclonal mouse IgG, anti-rat IgG kappa; L. Arnold). PA3-795.4.16 was isolated from culture supernatant by crude ammonium sulfate precipitation (50%), followed by dialysis in low osmolarity buffer at pH 5.5. All other antibodies were either used as culture supernatant or were isolated from ascites using sequential ammonium sulfate fractionation. Purified antibodies were stored in the cold at a concentration $\geq 5$ mg/ml in phosphate-buffered saline at pH7.4 following filter sterilization. Biotin and fluorescein conjugation were performed by standard methods. Fluorescein-conjugated goat anti-rat IgG Fc and goat anti-mouse IgG antisera were obtained from Rockland, Inc. (Gilbertsville, PA). Phycoerythrin-conjugated Streptavidin was obtained from Fisher Biotech, Inc.

### Flow Cytometry

Cell suspensions from thymus lobes were prepared by teasing gently with needles and then analyzed for phenotype on an EPICS C cytofluorograph as previously described (McDuffie et al., 1988). Staining protocols are outlined in each figure legend.

### Fetal Thymus Organ Culture

Cultures were performed essentially as previously described (Born et al., 1987). Briefly, thymus lobes were washed free of maternal blood in cold Hank's balanced salt solution (BSS) and placed on 0.45-$\mu$m Millipore filter strips supported by gelatin sponges. Five to 14 lobes per well were cultured in six-well tissue-culture plates with 3 ml of modified Mishell-Dutton medium (Mishell and Dutton, 1967) containing 10% heat-inactivated fetal bovine serum to avoid potential antibody-mediated complement activation. Thymus lobes were bathed twice daily with the medium containing PBS (untreated control) or with medium containing antibody at 100 $\mu$g/ml were cultured for 3 or 6–7 days. Medium and antibody were changed on day 3–4 for longer culture periods.

### ACKNOWLEDGMENTS

This work was supported by BRSG-05357 from the Biomedical Research Grant Program, NIH, and NIH fellowship AI-07840-02 (W.T.G.).

*(Received May 6, 1993)*

*(Accepted September 16, 1993)*

### REFERENCES

Bhattacharya A., Dorf M.E., and Springer T.A. (1981). A shared alloantigenic determinant in Ia antigens encoded by the I-A and I-E subregions: Evidence for I region gene duplication. J. Immunol. **127:** 2488–2495.

Born W., McDuffie M., Roehm N., Kushnir E., White J., Thorpe D., Kappler J., and Marrack P. (1987). Expression and role of the T cell receptor in early thymocyte differentiation in vitro. J. Immunol. **138:** 999–1008.

Born W., Rathbun G., Tucker P., Marrack P., and Kappler J. (1986). Synchronized rearrangement of T-cell gamma and beta chain genes in fetal thymus development. Science **234:** 479–482.

Body A.W., Wawryk S.O., Burns G.F., and Fecondo J.V. (1988). Intercellular adhesion molecules 1 (ICAM-1) has a central role in cell-cell contact-mediated immune mechanisms. Proc. Nat. Acad. Sci. USA **85:** 3095–3099.

Ceredig R., Lowenthal J.W., Nabholz M., and MacDonald H.R. (1985). Expression of interleukin-2 receptors as a differentiation marker on intrathymic stem cells. Nature **314:** 98–100.

Ceredig R., MacDonald H.R., and Jenkinson E.J. (1983). Flow microfluorometric analysis of mouse thymus development in vivo and in vitro. Eur. J. Immunol. **13:** 185–190.

Davignon D., Martz E., Reynolds T., Kurzinger K., and Springer, T.A. (1981). Monoclonal antibody to a novel lymphocyte function-associated antigen (LFA-1): Mechanism of blockade of T lymphocyte-mediated killing and effects on other T and B lymphocyte functions. J. Immunol. **127:** 590–595.

Fine J.S., and Kruisbeek A.M. (1991). The role of LFA-1/ICMA-1 interactions during murine T lymphocyte development. J. Immunol. **147:** 2852–2859.

Fowlkes B.J., Schwartz R.H., and Pardoll D.M. (1988). Deletion of self-reactive thymocytes occurs at a CD4+8+ precursor stage. Nature **334:** 620–623.

Golde W.T., McDuffie M., Kappler J., and Marrack P. (1990). Identification of a new cell surface glycoprotein with accessory function in murine T cell responses. J. Immunol. **144:** 804–810.

Hashimoto N., Nabholz M., MacDonald H.R., and Zubler R.H. (1986). Dissociation of interleukin 2-dependent and -independent B cell proliferation with monocloncal anti-interleukin 2 receptor antibody. Eur. J. Immunol. **16:** 317–320.

Haskins K., Kubo R., White J., Pigeon M., Kappler J., and Marrack P. (1983). The major histocompatibility complex-restricted antigen receptor on T cells. I. Isolation with a monoclonal antibody. J. Exp. Med. **157:** 1149–1169.

Kappler J.W., Roehm N., and Marrack P. (1987). T cell tolerance by clonal elimination in the thymus. Cell **49:** 273–280.

Kubo R.T., Born W., Kappler J.W., Marrack P., and Pigeon M. (1989). Characterization of a monoclonal antibody which detects all murine alpha beta T cell receptors. J. Immunol. **142:** 2736–2742.

Ledbetter J.A., and Herzenburg L.A. (1979). Xenogeneic monoclonal antibodies to mouse antigens. Immunol. Rev. **47:** 63–90.

Leo O., Foo M., Sachs D.H., Samelson L.E., and Bluestone J.A. (1987). Identification of a monoclonal antibody specific for a murine T3 polypeptide. Proc. Nat. Acad. Sci. USA **84:** 1374–1378.

Maryanski J.L., MacDonald H.R., Sordat B., and Cerottini J.-C. (1981). Cytolytic T lymphocyte orecursor cells in congenitally athymic C57BL/6 nu/nu mice: Quantitation, enrichment, and specificity. J. Immunol. **126:** 871–876.

McCarthy S.A., Kruisbeek A.M., Uppenkamp I.K., Sharrow S.O., and Singer A. (1988). Engagement of the CD4 molecule influences cell surface expression of the T-cell receptor on thymocytes. Nature **336:** 76–79.

McDuffie M., Roehm N., Kappler J.W., and Marrack P. (1988). Involvement of major histocompatibility complex products in tolerance induction in the thymus. J. Immunol. **141:** 1840–1847.

Mishell R., and Dutton R. (1967). Immunization of dissociated spleen cell cultures from normal mice. J. Exp. Med. **126:** 423–442.

Pardoll D.M., Fowlkes B.J., Lechler R.I., Germain R.N., and Schwartz R.H. (1987). Early genetic events in T cell development analyzed by in situ hybridization. J. Exp. Med. **165:** 1624–1638.

Prieto J., Takei F., Gendelman R., Christensen B., Biberfeld P., and Patarroyo M. (1989). MALA-2, mouse homologue of human adhesion molecule ICAM-1 (CD54). Eur. J. Immunol. **19:** 1551–1557.

Raulet D.H. (1985). Expression and function of interleukin-2 receptors on immature thymocytes. Nature **314:** 101–103.

Sharrow S.O., Mathieson B.J., and Singer A. (1981). Cell surface appearance of unexpected host MHC determinants on thymocytes from radiation bone marrow chimeras. J. Immunol. **126:** 1327–1335.

Shimonkevitz R.P., Husmann L.A., Bevan M.J., and Crispe I.N. (1987). Transient expression of IL-2 receptor precedes the differentiation of immature thymocytes. Nature **329:** 157–159.

Smith L. (1987). CD4+ murine T cells develop from CD8+ precursors in vivo. Nature **326:** 798–800.

